# First person – Robert Van Sciver

**DOI:** 10.1242/dmm.052116

**Published:** 2024-10-14

## Abstract

First Person is a series of interviews with the first authors of a selection of papers published in Disease Models & Mechanisms, helping researchers promote themselves alongside their papers. Robert Van Sciver is first author on ‘ A prioritization tool for cilia-associated genes and their *in vivo* resources unveils new avenues for ciliopathy research’, published in DMM. Robert is a postdoc in the lab of Tamara Caspary at Emory University School of Medicine, Atlanta, GA, USA, investigating primary cilia biology, in particular the ciliary mechanisms that drive kidney cyst formation.


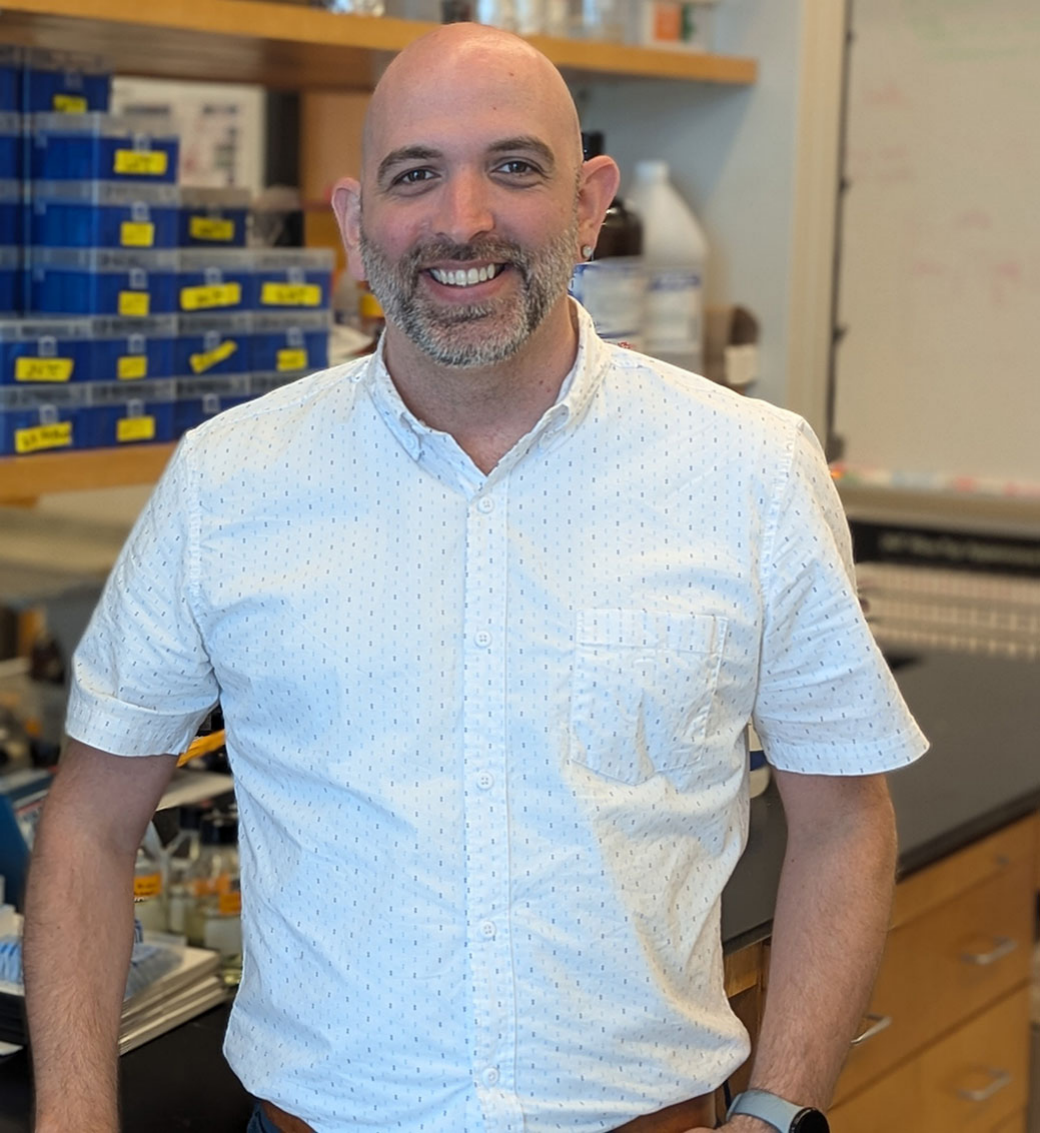


**Robert Van Sciver**

**Who or what inspired you to become a scientist?**

My parents, Bob and Debbie, inspired me to become a scientist by nurturing my naturally inquisitive nature. They continuously encouraged my questions of *why* and *how* rather than hushing me, even when my questions may have been annoying. In addition, my best friend's late mother, Suman Koura, also pushed me to further my education and career, and I owe it to her for where I am today.

**What is the main question or challenge in disease biology you are addressing in this paper? How did you go about investigating your question or challenge?**

There are many landmark studies identifying proteins that localise to primary cilia, yet each of these studies is a silo of what has been found. A major challenge, especially to people new to the field of cilia biology, is learning about these different proteins from all these different silos. In our paper, we have taken the grains from these silos and mixed them together so that we can get a more complete inventory of the proteins found in primary cilia, as well as a broader picture of the functions of these proteins, where they are expressed, and what mouse and zebrafish resources are available for their study.

Our paper establishes a resource for the scientific community to easily access information on genes they may want to study

**How would you explain the main findings of your paper to non-scientific family and friends?**

Our paper establishes a resource for the scientific community to easily access information on genes they may want to study. You can think of each gene as a book in the library, each with its own unique story, categorisation and characters. Sometimes the library doesn't have the book that you're looking for – maybe they never had it to begin with or someone returned it to the wrong shelf so you can't find it. Perhaps you can find the book at a neighbouring library though. We've taken the books from all these libraries and combined them into a single library that makes sure the book you're looking for will be readily available and easily accessible.

**What are the potential implications of these results for disease biology and the possible impact on patients?**

The resource we have prepared will be a boon to researchers studying rare diseases known as ciliopathies, caused by defects in primary cilia structure or function. As more and more large datasets are generated, having a resource like this to cross-reference hits will allow people to understand more about what is known about their ciliary genes of interest.

**Figure:**
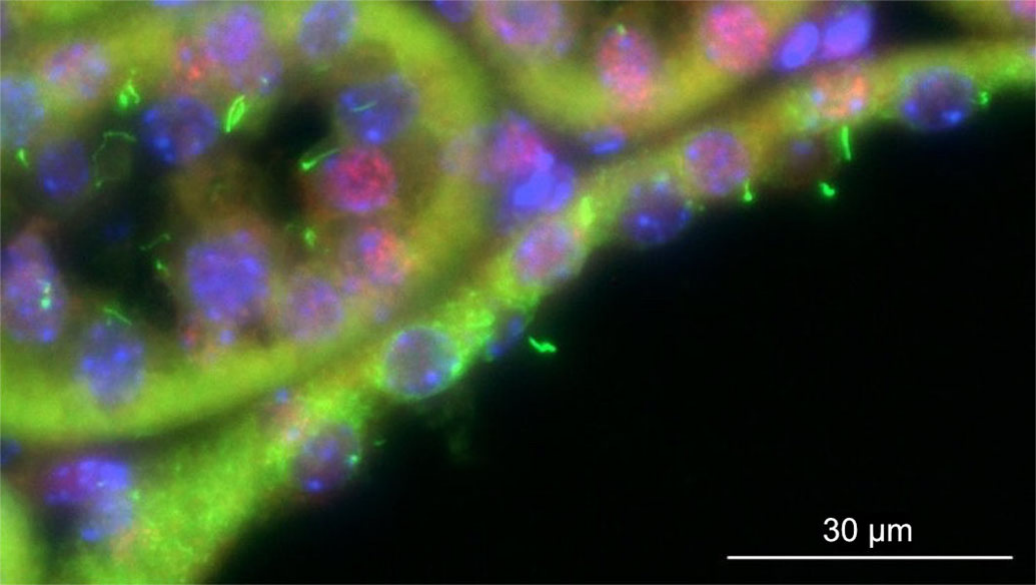
**Immunostaining of a kidney cyst in a mouse with an *Arl13b* mutation, preventing its detection in primary cilia.** Acetylated tubulin stains primary cilia in green, ARL13B staining is in red, and nuclei are stained blue.

**Why did you choose DMM for your paper?**

The Resources & Methods article type was the perfect fit for the Excel document we have assembled. In addition, while ciliopathies are typically rare, DMM has a broad readership, allowing this work to reach a wider audience. Lastly, I have always been a huge fan of and advocate for Open Access publishing.

**Given your current role, what challenges do you face and what changes could improve the professional lives of other scientists in this role?**

I am a postdoctoral fellow, and I recognise that while this role is often seen as underpaid, it's also a role of immense privilege. Many postdoc positions do not come with relocation assistance at a time that is often most critical. Graduate student stipends are already low, so your savings at the end of your PhD are often non-existent. Then you often move to a new city/state/country to begin your postdoc with limited resources – apartment application fees, security deposit, utilities. These all add up very quickly. I am fortunate to have had a support network in place that these were not issues for me, but that isn't the case for many.

**What's next for you?**

I'm currently on the academic job market. I plan to launch my independent lab in 2025, using genetic mouse models to investigate ciliary mechanisms that drive polycystic kidney disease.

**Tell us something interesting about yourself that wouldn't be on your CV**

I love to cook. I try around one new recipe each week and have a collection of around 1600 recipes in my recipe app, Paprika.

## References

[DMM052116C1] Van Sciver, R. E. and Caspary, T. (2024). A prioritization tool for cilia-associated genes and their *in vivo* resources unveils new avenues for ciliopathy research. *Dis. Model. Mech.* 17, dmm052000. 10.1242/dmm.052000 39263856 PMC11512102

